# Supplementary material for: The interaction of Escherichia coli O157 :H7 and Salmonella Typhimurium flagella with host cell membranes and cytoskeletal components
Source: Microbiology (Reading). 2020 Sep 4;166(10):947–65. doi: 10.1099/mic.0.000959 (PMC7660914; doi:10.1099/mic.0.000959)
Supplement: Supplementary material 1 [file mic-166-947-s001.pdf]

Supplementary Information

Figure S1. Confocal microscopy of intracellular interactions of H7 flagella with host cells. Confocal z-slices of *E. coli* O157:H7 TUV93-0 flagella (green) interacting with actin (red). Nuclei staining is cyan. **(A1)** XY projection of TUV93-0 interacting with EBL cells, 3 h post infection. The micro-colony indicated with an arrow shows an H7 flagellum curling round A/E lesions. The labelled inset marks the XZ and YZ projections analysed in B1-B2. **(B1-B2)** Arrows point to specific regions within individual XZ **(B1)** and YZ **(B2)** slices in which H7 flagellum is inside a region of actin staining. **(C1)** Top panel shows an XY projection of the whole field from which the images in Fig. 1A were taken. Middle panels show Fig. 1A XZ and YZ projections, free of actin/flagellum co-incidence labelling. Lower panels show actin-H7 flagellum co-incidence (yellow) across individual z-slices in XZ and YZ planes. Scale bars = 5 µm.

(Figure on next page)

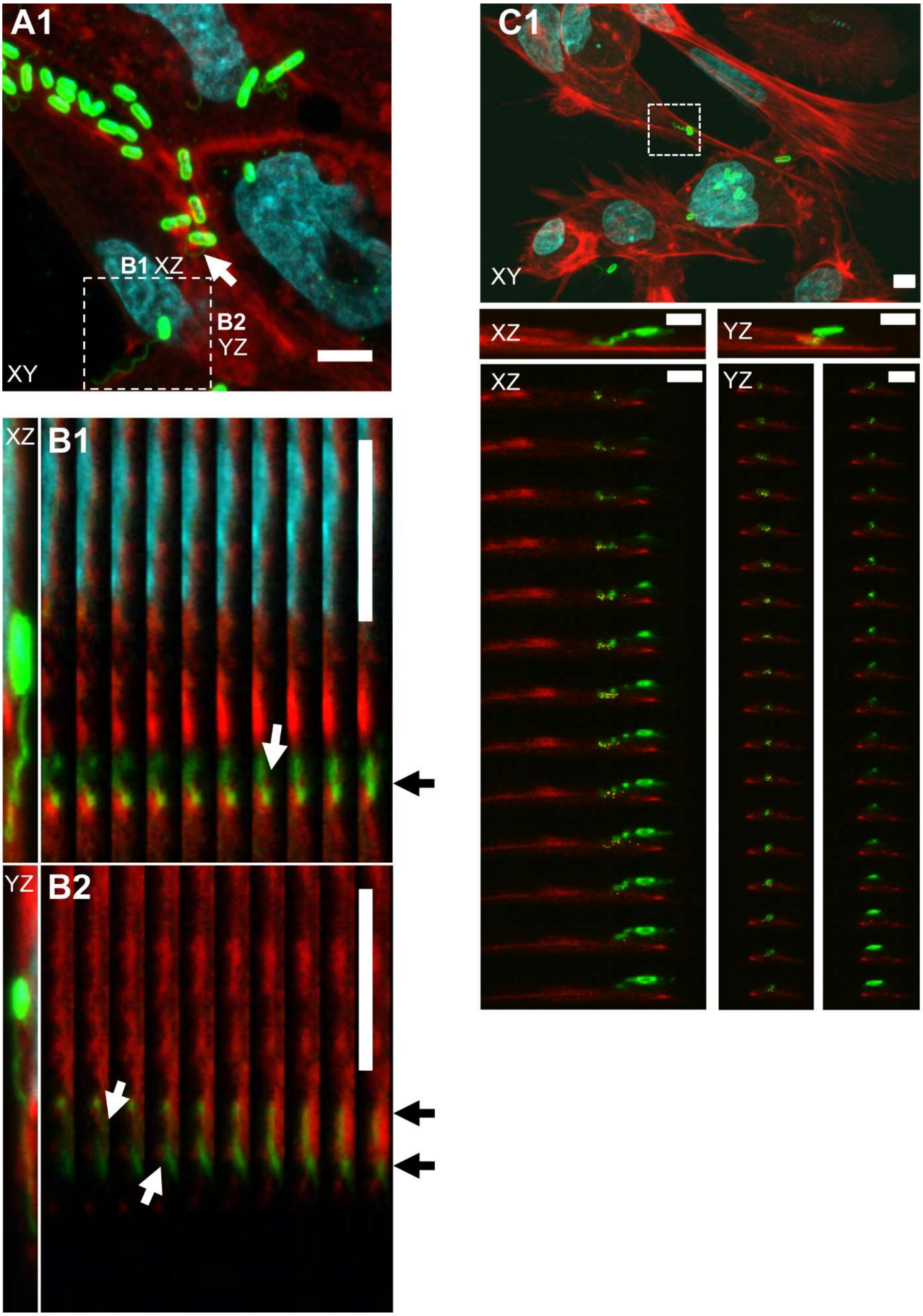
A1
B1 XZ
B2
YZ
XY
XZ
B1
YZ
B2
C1
XY
XZ
YZ
XZ
YZ

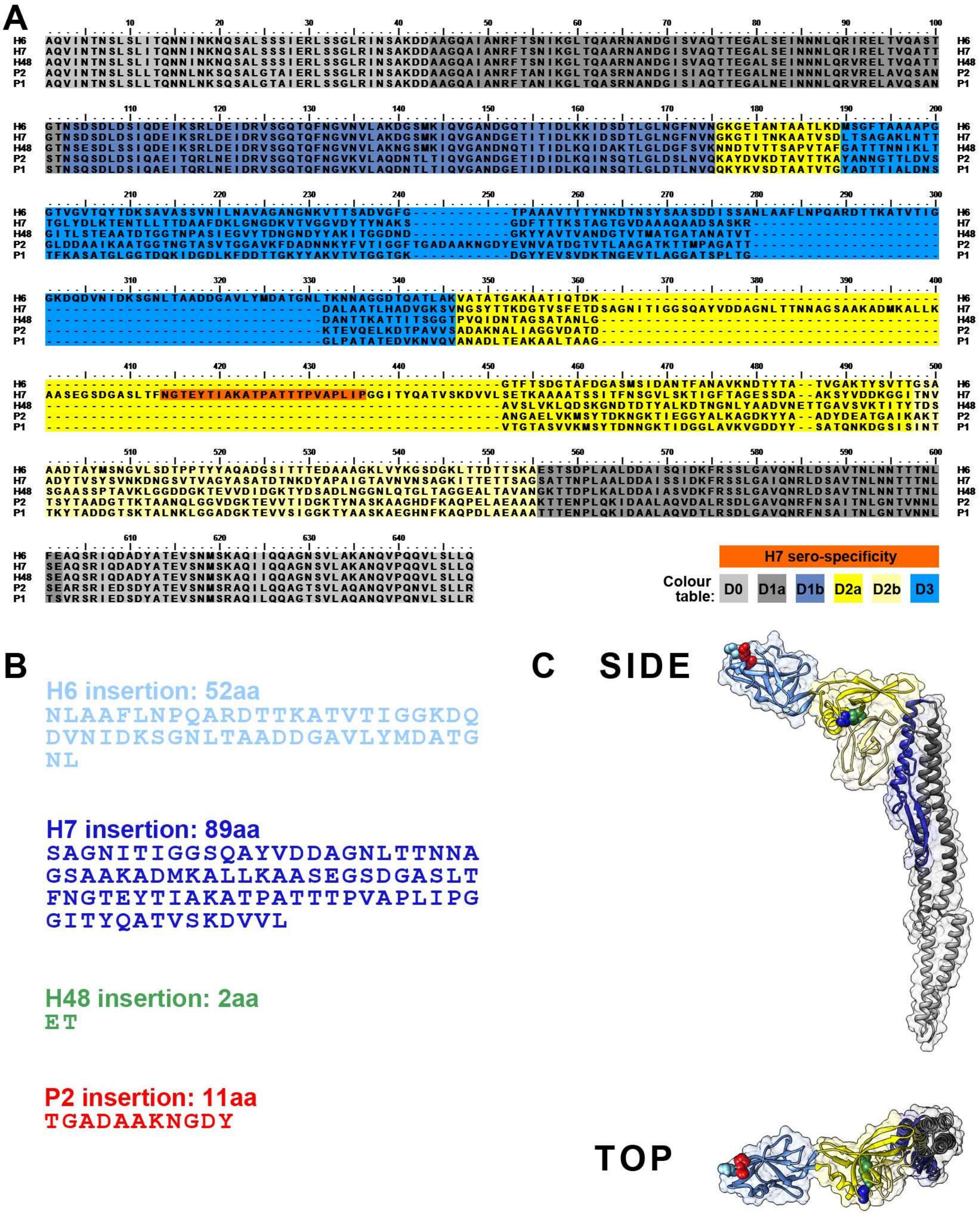

Figure S2. Protein sequence and structural differences between H6, H7, H48, P1 and P2 flagellins. (A) Pairwise structural alignments with P1 flagellin (PDB: 1UCU) were generated using the FFAS03 server from its PDB database. Alignments were then stitched together and presented in BioEdit v 7.2.0. Amino acids are coloured by structural domain, as defined by Yonekura *et al.*[1] and outlined in the colour table. This model is partially validated by the presence of the H7-serospecific region in the H7-specific structural insertion[2], (orange). **(B)** Amino acid sequences modelled to be structurally dissimilar to the P1 structural template, as determined by the alignment in (A). **(C)** Location of structural insertions in P1 flagellin indicated by spherically presented side-chains, colour-coded as in (B). The P1 structural model is presented coloured by structural domain as in (A), with top and side views, in USCF Chimera v 1.8[3].

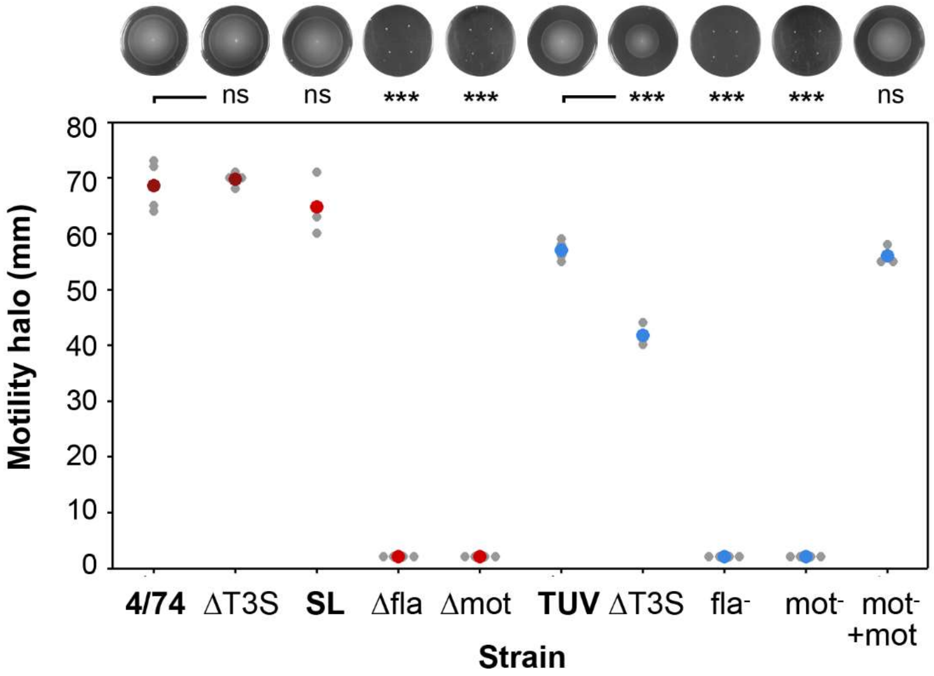

Figure S3. Motility *S.* Typhimurium and *E. coli* O157:H7 strains used in haemolysis assays. Motility of strains used in Fig. 6 was measured by the radius of growth after inoculation into 0.3% (w/v) LB agar at RT for 36 h. SL= SL1344. The top panel shows representative motility plates, and the bottom panel shows radii measurements from point of inoculation of four biological replicates. Statistical analyses of these are presented as 2-tailed homoscedastic students T-tests (p≤0.0001 = ***) against WT (4/74 or TUV93-0).

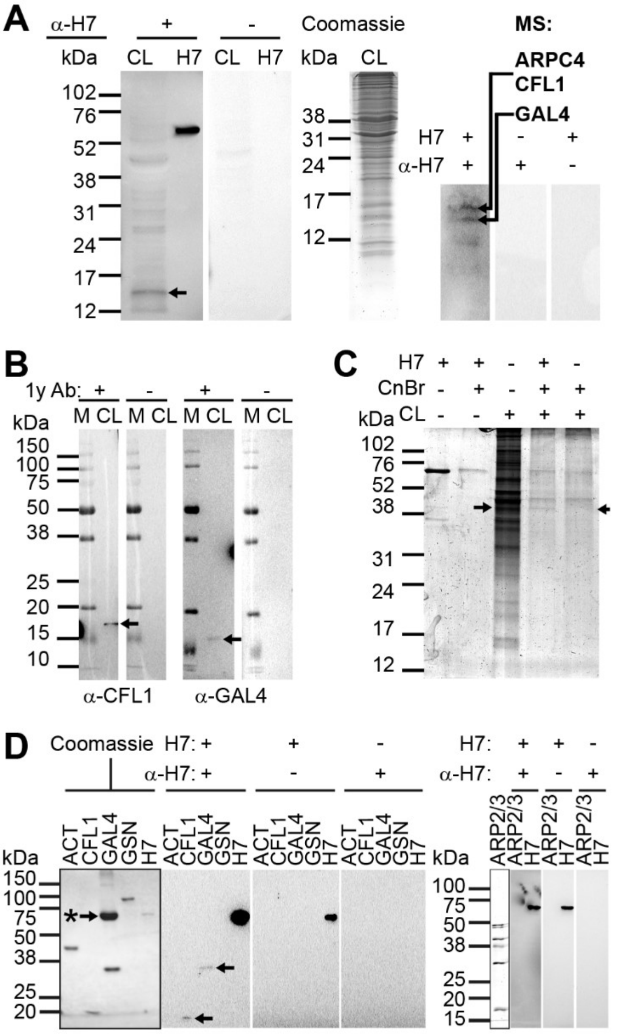

Figure S4. Binding substrates for H7 flagella present in bovine terminal rectum epithelial lysates. (A) Far-Western blot of primary bovine rectal epithelial cell lysates (CL) probed with H7 flagella and H7-specific antibodies. Left-hand blots show one reacting band (arrow) resolved further (right) and the bands indicated were identified as Arp2/3 complex sub-unit 4 (ARPC4), cofilin-1 (CFL1) and galectin-4 (GAL4) by mass-spectrometry (MS). **(B)** Western-blots of primary bovine rectal epithelial cell lysates (CL) confirmed the presence of CFL1 and GAL4 between 15-20 kDa with α-CFL1 and α-GAL4 antibodies (arrows, Table S3). The size discrepancy of GAL4 can be explained by its degradation or processing within cell lysates, as has been observed previously[4,5]. **(C)** Pull-down of bovine β-actin from primary bovine rectal epithelial cell lysates (CL) by H7 flagella cross-linked to CnBr-activated Sepharose beads. The cell lysates were prepared by freeze-thawing. Pre-cleared empty beads were used as a negative control (final lane). Arrows indicate 38-50 kDa protein bands in the coomassie-stained gel that were excised and identified as β-Actin (ACTB1) by MS. **(D)** Far-Western blots of 1 μg purified human βγ-actin (ACT), recombinant human CFL1, recombinant human GAL4 and purified gelsolin (GSN) from bovine plasma with negative control blots on the left, and 1 μg purified ARPC4 from bovine brain with detection controls on the right, probed with H7 flagella and H7-specific antibodies as indicated. The ~60 kDa band indicated with an arrow marked with an (*) is 0.1% (w/v) BSA, a carrier protein for GAL4. 0.5 μg H7 was loaded as a positive detection control. Detected bands are indicated with arrows. Marker lane (M) sizes are in kDa on the left throughout.

Movie S1. Co-localisation of *E. coli* O157:H7 flagella with phalloidin on a bovine terminal rectal epithelial cell. A rotating three-dimensional projection of a confocal micrograph that has captured *E. coli* O157:H7 (green) flagella coincident with bovine primary rectal epithelial actin (red, Fig. 1A). Co-incident staining of actin and O157:H7 is shown in yellow. The 3D projection was made and presented using NIH ImageJ software.

Movie S2. Tomographic slice of the flagellated *S.* Typhimurium within an epithelial cell. A view up and down through the 3D projection of the tomogramic slice shown in figure 5A1, zooming in to the inset shown in figure 5A2. An anisotropic diffusion filter was applied to reduce noise.

Movie S3. Tomographic slice of the flagellated *S.* Typhimurium inside a membrane ruffle. A view up and down through the 3D projection of the tomogramic slice shown in figure 5B1, zooming in to the inset shown in figure 5B2. An anisotropic diffusion filter was applied to reduce noise.

Movie S4. Tomographic slice of the flagellated *S.* Typhimurium at the point of induced uptake into an epithelial cell shown in figure 5C. A view up and down through the 3D projection of the tomogramic slice shown in figure 5C1, with segmentation analysis of host cell membranes shown in red, zooming in to the inset shown in figure 5C2. An anisotropic diffusion filter was applied to reduce noise.

Table S1. Plasmids used or constructed in this study.

Plasmid	Relevant features	Source
pAJR145	Amp^r, constitutively expressed *egfp*	[6]
pIB307	Chl^r, Kan^r, T^oC^s replication (28ºC), single copy	[7]
pTOF25	Chl^r, Kan^r, T^oC^s replication (28ºC), single copy	[8]
pEBW6	pIB307; H7downB flank; H6 CDS; H7up flank	This study
pEBW7	pIB307; H7downB flank; H7 CDS; H7up flank	This study
pEBW5	pWSK29 $fliC_{H48}$ from *E. coli* K-12 MG1655	[9]
pTOF*motA*	pTOF25; *motA* N flank; Kan^r; *motA* C flank	This study

Table S2. Primer pairs used in this study. Annealing temperatures used indicated in bold.

Product	Primer Name	Primer Sequence	Source
H7up flank	5'H7upF.SacI	AAGAGCTCTATTGCCTGTGCCACTTCAC	[9]
**55°C**	3'H7upR.BamHI	AAGGATCCTAACTGAGACTGACGGCAAC	[9]
H7downB flank	H7downF3.BamHI	GGGGATCCCACCCGTCGGCTCAATCG	This study
**55°C**	3'H7downR.SalI	AAGTCGACTTCGTATCGTCTCTGGTGGT	This study
H7 CDS	3'NtH7F2.BamHI	GGGGATCCCAATACGTAATCAACGACTTGC	This study
**58°C**	5'CtH7R.BamHI	AAGGATCCTTAACCCTGCAGCAGAGACAG	This study
H6 CDS	3'NtH6F2.BamHI	GGGGATCCCAAAACGTAATCAACGACTTGC	[10]
**58°C**	JTH11fliC.R.BamHI	CCGGATCCCTAACCCTGCAGCAGAGACAG	[10]
sacB	SacB 5’	GCAACTCAAGCGTTTGCGAAA	[11]
**55°C**	SacB 3’	GGCTTGTATGGGCCAGTTAAG	[11]
O157 specific	O157F	CGGACATCCATGTGATATGG	[12]
**55°C**	O157R	TTGCCTATGTACAGCTAACC	[12]
pIB307 insert	pIB073-screen.F	CCTGTCCTACGAGTTGCATG	[13]
**60°C**	pIB073-screen.R	GACTCCTGCATTAGGAAGCA	[13]
FliC locus	5'H7upF.SacI	AAGAGCTCTATTGCCTGTGCCACTTCAC	This study
**55°C**	3'H7downR.SalI	AAGTCGACTTCGTATCGTCTCTGGTGGT	This study
MotA N flank	No-motA	TTGCTGGTCTCGGTACCCGGG<br>CGACAACATTAGCGGCACTGACTC	This study
**50°C**	Ni-motA	CGCTCTTGCGGCCGCTTGGAACGG<br>GACATCATCCTTCCACTGTTGACC	This study
MotA C flank	Co-motA	TCCCATTCGCCACCGGTCGAC<br>CACGCTGTCACCTCGGTTCGGCTG	This study
**50°C**	Ci-MotA	CCGTTCCAAGCGGCCGCAAGAGCG<br>ATGTGCGTGCGGTGAAAAATCCGC	This study

Table S3. Antibodies and stains used in this study.

Primary Antibody	Details	Source
α-H6	polyclonal rabbit IgG	Mast Assure
α-H7	polyclonal rabbit IgG	Mast Assure
α-FliC (P1)	polyclonal rabbit IgG	Ariel Blocker Lab, University of Bristol
α-Hi (P1)	polyclonal rabbit IgG	Mast Assure
α-H2 (P2)	polyclonal rabbit IgG	Difco
α-P1+P2	polyclonal rabbit IgG	Difco
α-O157:H7	polyclonal rabbit IgG	Mast Assure
α-O157	monoclonal mouse IgG	Abcam
α-O4	polyclonal rabbit IgG	Mast Assure
α-cofilin-1	monoclonal mouse IgG	AbDSeroTec
α-galectin-4	polyclonal goat IgG	R&D
**Secondary Antibody**	**Details**	**Source**
α-rabbit IgG-HRP	polyclonal goat IgG	R&D
α-goat IgG-HRP	polyclonal rabbit IgG	R&D
α-mouse IgG-HRP	polyclonal donkey IgG	R&D
α-rabbit IgG-FITC	polyclonal goat IgG	Sigma
α-rabbit IgG-FITC	polyclonal goat IgG	R&D
α-rabbit IgG-10nm gold	polyclonal goat IgG	British Biocell Intl.
α-rabbit IgG- AlexaFluor568	polyclonal goat IgG	Molecular probes
α-mouse IgG- AlexaFluor568	polyclonal goat IgG	Molecular probes
α-rabbit IgG- AlexaFluor594	polyclonal goat IgG	Molecular probes
**Stains**		**Source**
Phalloidin-Texas Red		Invitrogen
Phalloidin-AlexaFluor588		Molecular Probes
Phalloidin-AlexaFluor647		Invitrogen
Phalloidin-FITC		Molecular Probes
Phalloidin-TRITC		Sigma
DAPI		Merck
Wheat-germ agglutinin-Texas Red		Invitrogen

Supplementary references

1. **Yonekura, K., Maki-Yonekura, S. & Namba, K.** Complete atomic model of the bacterial flagellar filament by electron cryomicroscopy. *Nature* **424**, 643–650 (2003).

2. **Kwang, J., Wilson, R., Yang, S. & He, Y.** *Mapping of the H7-Serospecific Domain of Escherichia coli Flagellin. CLINICAL AND DIAGNOSTIC LABORATORY IMMUNOLOGY* **3**, (1996).

3. **Pettersen, E. F. *et al.*** UCSF Chimera--a visualization system for exploratory research and analysis. *J. Comput. Chem.* **25**, 1605–12 (2004).

4. **Leffler, H., Masiarz, F. R. & Barondes, S. H**. Soluble lactose-binding vertebrate lectins: a growing family. *Biochemistry* **28**, 9222–9229 (1989).

5. **Oda, Y. *et al.*** Soluble lactose-binding lectin from rat intestine with two different carbohydrate-binding domains in the same peptide chain. *J. Biol. Chem.* **268**, 5929–5939 (1993).

6. **Roe, A. J. *et al.*** Co-ordinate single-cell expression of LEE4- and LEE5-encoded proteins of Escherichia coli O157:H7. *Mol. Microbiol.* **54**, 337–352 (2004).

7. **Blomfield, I. C., Vaughn, V., Rest, R. F. & Eisenstein, B. I.** Allelic exchange in Escherichia coli using the Bacillus subtilis sacB gene and a temperature-sensitive pSC101 replicon. *Mol. Microbiol.* **5**, 1447–1457 (1991).

8. **Merlin, C., McAteer, S. & Masters, M.** Tools for characterization of Escherichia coli genes of unknown function. *J. Bacteriol.* **184**, 4573–81 (2002).

9. **Rossez, Y. *et al.*** Flagella interact with ionic plant lipids to mediate adherence of pathogenic Escherichia coli to fresh produce plants. *Environ. Microbiol.* **16**, (2014).

10. **Mahajan, A. *et al.*** An investigation of the expression and adhesin function of H7 flagella in the interaction of Escherichia coli O157: H7 with bovine intestinal epithelium. *Cell. Microbiol.* **11**, 121–137 (2009).

11. **Xu, X. *et al.*** Lysogeny with Shiga Toxin 2-Encoding Bacteriophages Represses Type III Secretion in Enterohemorrhagic Escherichia coli. *PLoS Pathog.* **8**, e1002672 (2012).

12. **Paton, A. W. & Paton, J. C.** Detection and characterization of shiga toxigenic escherichia coli by using multiplex PCR assays for stx1, stx2, eaeA, enterohemorrhagic E. coli hlyA, rfb(O111), and rfb(O157). *J. Clin. Microbiol.* (1998).

13. **Flockhart, A. F. *et al.*** Identification of a novel prophage regulator in Escherichia coli controlling the expression of type III secretion. *Mol. Microbiol.* **83**, 208–223 (2012).
